# Mechanical Energy Expenditure-based Comfort Evaluation Model for Gesture Interaction

**DOI:** 10.1155/2018/9861697

**Published:** 2018-12-30

**Authors:** Wenjie Wang, Xiansheng Qin, Chen Zheng, Hongbo Wang, Jing Li, Junlong Niu

**Affiliations:** School of Mechanical Engineering, Northwestern Polytechnical University, Xi'an 710072, China

## Abstract

As an advanced interaction mode, the gesture has been widely used for the human-computer interaction (HCI). The paper proposes a comfort evaluation model based on the mechanical energy expenditure (MEE) and the mechanical efficiency (ME) to predict the comfort of gestures. The proposed comfort evaluation model takes nineteen muscles and seven degrees of freedom into consideration based on the data of muscles and joints and is capable of simulating the MEE and the ME of both static and dynamic gestures. The comfort scores (CSs) can be therefore calculated by normalizing and assigning different decision weights to the MEE and the ME. Compared with the traditional comfort prediction methods based on measurement, on the one hand, the proposed comfort evaluation model makes it possible for providing a quantitative value for the comfort of gestures without using electromyography (EMG) or other measuring devices; on the other hand, from the ergonomic perspective, the results provide an intuitive indicator to predict which act has the higher risk of fatigue or injury for joints and muscles. Experiments are conducted to validate the effectiveness of the proposed model. According to the comparison result among the proposed comfort evaluation model, the model based on the range of motion (ROM) and the model based on the method for movement and gesture assessment (MMGA), a slight difference can be found due to the ignorance of dynamic gestures and the relative kinematic characteristics during the movements of dynamic gestures. Therefore, considering the feedback of perceived effects and gesture recognition rate in HCI, designers can achieve a better optimization for the gesture design by making use of the proposed comfort evaluation model.

## 1. Introduction

Nowadays, as an advanced interaction mode, the gesture has been widely used for the human-computer interaction, because it is more natural, convenient, and efficient than the traditional input modes, such as mouse, keyboard, handle, etc. Through the application of gesture-based interaction technique, the computer translates the gestures into control commands. Especially in the domains of video games and smart phones, the gesture-based interaction mode achieves great success by virtue of the favorable users' experiences. By means of the gestures, users can manipulate the virtual objects [1, 2], acquire the remote targets [3, 4], select the menus [5–7], type the text, etc. Therefore, the gesture interaction has attracted widespread research interests, such as biomechanical modeling, comfort evaluation, gesture design, gesture recognition and gesture-based HCI. As a result, on the one hand, the comfort of gestures plays a significant role to estimate the load of muscles in order to ensure operators' safety and prevent them from getting injured. On the other hand, in the gesture design, the ergonomic levels of gestures directly affect operating comfort, convenience, and efficiency, and comfortable gestures result in a good matching between operators and their muscles, so that their fatigue can be reduced and working hours can be extended. Nowadays, the comfort evaluation in gesture design has attracted attention from both academia and industry. However, current studies do not fully account for the complex reality of human gesture. The authors propose a mechanical energy expenditure-based model which considers the influences of both static and dynamic gestures to help designers evaluate the comfort of gestures during gesture interaction process. Firstly, the proposed comfort evaluation model provides designers with a quantitative value for the comfort of gestures without using EMG or other measuring devices; secondly, from the ergonomic perspective, the results obtained from the proposed comfort evaluation model can be regarded as an intuitive indicator to predict which act has the higher risk of fatigue or injury for joints and muscles, so as to reduce operators' fatigue and extend their working hours.

## 2. Related Works

In the context of gesture interaction, the comfort is defined as the level of an operator's well-being when he/she interacts with his/her working environment. However, this level is difficult to detect or measure because it is affected by the operator's subjective feelings and individual judgment [8]. Currently, there are mainly four classes of solutions to the problem of comfort evaluation: (1) model based on the ROM, (2) model based on the ROM and movement data, (3) methods based on software tools, and (4) measurement based on devices or sensors.

For the comfort evaluation model based on the ROM of joints, various comfort evaluation models, such as JAI [9], RULA [10, 11], LUBA [12], REBA [13], OCRA, NERPA [14],UNE-EN 1005 [14], etc., have been developed, which have been applied on the ergonomic design [15–20] in HCI. Above models provide semiquantitative indicators because only static gestures are taken into consideration. However, the operator's comfort always varies with the change of joint angles during the task execution process. Thus, the dynamic gesture is another key factor which influences the operator's comfort. In order to evaluate the dynamic gestures, current studies have proposed various comfort evaluation models [21–25]. Andreoni et al. [22] present a MMGA model for the ergonomic ranking of motor tasks. The model is derived from the LUBA model that is evaluated by the static gesture. Weede et al. [23] presents the criteria for skill evaluation and ergonomic conditions with assigned weights and proportions. However, there is a lack of biomechanical information in the criteria. Keyvani et al. [25] use a digital human modeling (DHM) tool to evaluate the ergonomic risks. However, the tool cannot be modified as needed. Besides, software tools (e.g., JACK [16], CATIA [26], etc.) and measuring devices or sensors (e.g., EMG, motion capture, pressure distribution measurement system [27], etc.) have been developed to measure the comfort of gestures.

In general, current studies provide valuable experiences in the comfort evaluation. However, limitations still exist in the propositions previously reviewed.  Table 1 shows the advantages and limitations of each proposition. The ROM-based model evaluates the comfort of gestures based on static gestures. The comfort of gestures can be directly obtained from the calculation results of each model, which are very convenient and designers do not need many professional skills, but the dynamic gestures and the biomechanical information are not considered by the ROM-based model. Moreover, the indicator for evaluating the comfort obtained from the ROM-based model is discontinuous, so it cannot provide a precise quantitative value. The ROM and movement data-based model retains the advantages of ROM-based model. Moreover, it takes both static and dynamic gestures into consideration, but the model is complex, which requires certain professional skills. Both of the software-based method and the sensors-based method can provide designers with precise quantitative values, but their costs are very high and their applications also need more professional skills.

After analyzing the advantages and limitations of previous studies, the authors propose a comfort evaluation model based on the MEE to achieve a better optimization for the gesture design. As an important biological characteristic, energy expenditure has been studied by researchers in the domain of ergonomics. The accurate muscle energy expenditure is essential for evaluating and analyzing the comfort of gestures. Umberger et al. [28] present a model of human muscle energy expenditure for predicting the thermal and mechanical energy liberation by simulating muscle contractions. However, this model has not been applied to ergonomic analysis. Kistemaker et al. [29] present an energy expenditure model, but the application of the model on comfort of gestures is not mentioned in the study. Battini et al. [30] apply the motion energy system to estimate the energy expenditure for predicting the ergonomic level. However, this study believes that metabolic energy expenditure depends on the gender, body weight, load weight, movement position of arms, velocity, duration, etc., rather than the muscle contraction. Wang et al. [31] propose an approach to relieve operator's fatigue in an assembly line based on the metabolic energy expenditure. However, the model only focuses on the static gestures.

The paper focuses on the comfort evaluation model based on the MEE to predict the comfort of gestures. By using the real musculoskeletal data and mass-inertia characteristics, the biomechanical model of upper limb is constructed to simulate the human gestures and calculate the MEE and ME of gestures, in which both static and dynamic gestures are taken into account. The comfort scores can be therefore calculated by normalizing and assigning different decision weights to the MEE and the ME, which provide a quantitative and intuitive indicator for evaluating the comfort of gestures. Finally, the experiments are conducted to validate the effectiveness of the proposed model. Next section presents the details of the proposed comfort evaluation model.

## 3. Comfort Evaluation Model

### 3.1. Musculoskeletal Kinematics Modeling

**Figure 1:**
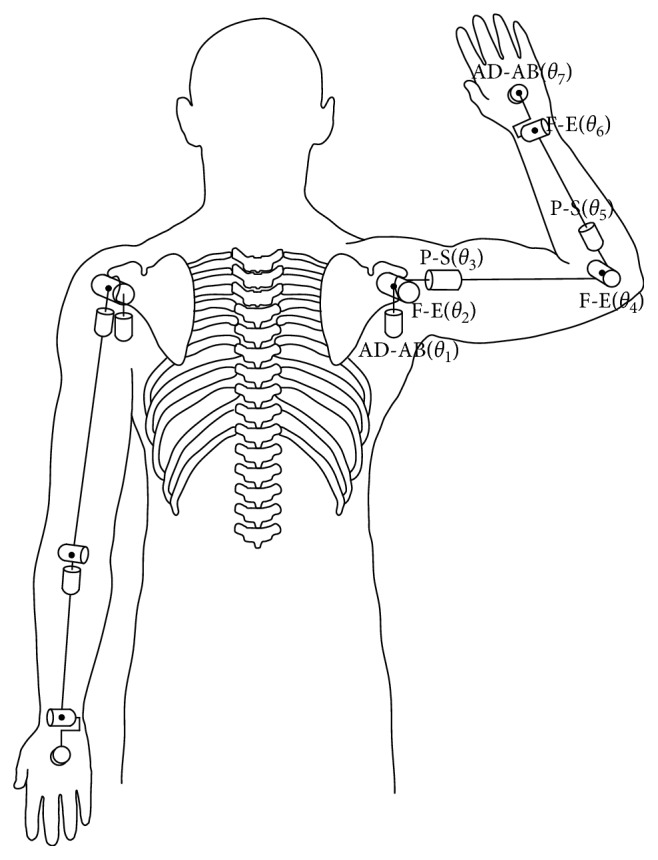
Human upper limb model.

Biomechanical characteristics of humans should be taken into consideration so that the model can accurately simulate the human gestures. One hypothesis which is adopted in the proposed model is that the upper limb can be simplified as multirigid body hinge structure [32]. In order to avoid the complexity of human body, the authors simplify the upper limb as the mechanical model with three segments and seven degrees of freedom. In Figure 1, the joint angles of model are expressed by *θ*_*i*_=(*θ*_1_, *θ*_2_, *θ*_3,_*θ*_4_, *θ*_5_, *θ*_6_, *θ*_7_), respectively. The F-E represents the flexion-extension of joint, the P–S represents the pronation-supination of joint, and the AD-AB represents the adduction-abduction of joint.

**Figure 2:**
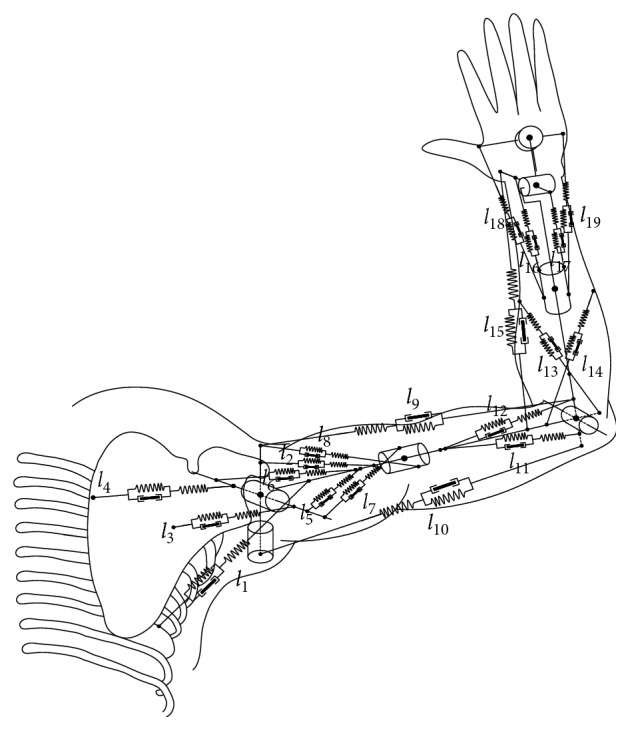
Upper limb musculoskeletal model.

According to the biomechanics, the muscles attach different skeletons and control the movements of skeletons around the joints [33]. Considering the biomechanical information related to the muscles of right upper limb, the authors propose an upper limb musculoskeletal model based on the human upper limb model presented previously, which consists of three segments, sixteen one-joint muscles, and three two-joint muscles (Figure 2). The model shows the relationship between the muscles and the joint motions; therefore it can accurately simulate the muscle contractions and the three-dimensional movements.  Table 2 shows the corresponding relationship between the muscles and the joint motions.

The muscle can shorten to produce a concentric contraction and lengthen to produce an eccentric contraction. In order to establish the musculoskeletal kinematic model, the kinematics of muscles and joints can be calculated by the following formula:

(1) $$\begin{array}{c} l\left(\theta_{i}\right)={\left(l_{1},l_{2},l_{3},\cdots ,l_{19}\right)}^{\text{T}}, \end{array}$$

where *l*_1_, *l*_2_, *l*_3_, ⋯, *l*_19_ are the lengths of the nineteen muscles, *θ*_*i*_(*i*=1, 2,…7) are the angles of the seven joints. Taking the derivative of formula (1), the contraction velocities of muscles can be obtained as

(2) $$\begin{array}{c} \dot{l}=D\dot{\theta }, \end{array}$$

where $\dot{l}$ and $\dot{\theta }$, respectively, are the vector of the velocities of the nineteen muscles and the vector of the angle velocities of the seven joints, respectively and *D* ∈ ℜ^19×7^ represents the Jacobian matrix from the joint space to the muscle space. According to the virtual work principle, the joint torques can be expressed as [34]

(3) $$\begin{array}{c} T_{\text{M}}=DF_{\text{M}}, \end{array}$$

where *T*_M_ represents the vector of the joint torques generated by the muscles, *F*_M_ represents the vector of the tensile forces of muscles, and *D* is the moment arms of muscles. According to the kinematics, the Jacobian matrix equals the moment arms of muscles.

### 3.2. Biomechanics Modeling

The relationship of force-velocity links the contraction velocities at which the muscles change their lengths to the forces generated by muscles. The contraction velocities of muscles can be obtained by the derivative of the muscles' lengths during the movements of dynamic gestures. The force-velocity relationship can be described by Hill's muscle model [35]:

(4) $$\begin{array}{c} \left(F_{\text{M}}+a\right)\left(\dot{l}+b\right)=\left(F_{0}+a\right)b. \end{array}$$

Thus, the tensile forces of muscles can be represented as follows:

(5) $$\begin{array}{c} F_{\text{M}}=\frac{F_{0}b-a\dot{l}}{\dot{l}+b}, \end{array}$$

where *F*_M_ is the vector of the tensile forces of muscles; $\dot{l}$ is the vector of the contraction velocities of muscles; *F*_0_ is the vector of the maximum isometric forces; *a* is the vector of the coefficients of contraction heat; *b* equals *av*_0_/*F*_0_; and *v*_0_ is the vector of the maximum velocities when *F*_M_ = 0. Based on the experiences and achievements of previous studies [36], the empirical constants can be calculated as follows:

(6) $$\begin{array}{c} a=0.25F_{0},\\ b=0.25v_{0}. \end{array}$$

According to the Newton–Euler formulation, the joint torque is affected by multiple factors, such as inertial force, centrifugal force, gravity force, muscle force, and the limit of ligaments to the joint. The relationship between the forces applied to the limbs and the resulting motions of the limb segments can be expressed by the following formula:

(7) $$\begin{array}{c} \tau =M\left(q\right)\ddot{q}+C\left(q\right){\dot{q}}^{2}+G\left(q\right)+DF_{\text{M}}+T^{\text{lig}}, \end{array}$$

where *τ* is the vector of joint torques; $q,\dot{q},\ddot{q}$ are the vectors of the joint coordinates, the velocities, and the accelerations, respectively; *M*(*q*) is the inertia matrix and $M\left(q\right)\ddot{q}$ is a vector of inertial torques; *C*(*q*) is the coriolis matrix and $C\left(q\right){\dot{q}}^{2}$ is the vector of coriolis torques; *G*(*q*) is the vector of gravity forces; *D* is the moment arms of muscles, *F*_M_ is the vector of muscle forces and *DF*_M_ is the vector of moment of muscle force; and *T*^lig^ is the vector of ligament torques. Frictions are not taken into consideration in the formula, because they are so small when compared with muscle forces.

### 3.3. Mechanical Energy Expenditure

In classical mechanics, work is the application of a force over a distance. The change in energy of an object is equal to the work done on the object. However, in biomechanics, the main topic of interests is not the total power or the work done on the body, but the MEE. The MEE of human movements is a worthy reference that contains rich biomechanical information. During the symmetric movements, the total work done on the body is zero, but the MEE is not zero. Generally, the muscles energy expenditure consists of the MEE and heat dissipation. Because the function of muscles is mainly to generate mechanical forces, the heat dissipation can be neglected during muscle contraction and the authors concentrate on a pure mechanical approach about human energy expenditure.

For the joint movements [29], the mechanical power of joints can be calculated as the sum of scalar product of the vector of joint torques and the angular velocities:

(8) $$\begin{array}{c} P=\overset{7}{\underset{i=1}{\sum }}\tau \dot{\theta }, \end{array}$$

where *P* represents the mechanical power of joints. The mechanical work of joints can be calculated as the sum of the integral of the product of joint torques and the angular velocities over the duration from *t*_1_ to *t*_2_:

(9) $$\begin{array}{c} W=\overset{7}{\underset{i=1}{\sum }}\int_{t_{1}}^{t_{2}}\tau \dot{\theta }\;dt. \end{array}$$

The muscle never works alone. The joint motions are controlled by the muscle forces generated by the agonist and antagonist that do the positive work and negative work, respectively. Thus, the MEE can be calculated as a sum of integral of the absolute values of positive and negative works of joints:

(10) $$\begin{array}{c} \text{MEE}=\overset{7}{\underset{i=1}{\sum }}\int_{t_{1}}^{t_{2}}\left(\left|\tau_{i}^{+}\dot{\theta }\right|+\left|\tau_{i}^{-}\dot{\theta }\right|\right)dt, \end{array}$$

where *τ*_*i*_^+^ and *τ*_*i*_^−^ are the joint torques generated by the agonist and antagonist muscles, respectively. However, the model is limited and cannot account for the MEE of static gestures, because the MEE equals zero when $\dot{\theta }$ equal zero.

Therefore, for dynamic gestures, the MEE is the sum of the integral of the sum of the absolute values of the power of the joint torques and the muscle forces, while for static gestures, the MEE is the sum of the absolute value of the product of the muscle forces of the agonist and antagonist and the duration [39]. The MEE can be optimized and expressed by the following formula:

(11) $$\begin{array}{c} \text{MEE}=\left\{\begin{array}{cc} \overset{7}{\underset{i=1}{\sum }}\overset{t_{2}}{\underset{t_{1}}{\int }}\left(\left|T_{i}^{+}\dot{\theta }\right|+\left|T_{i}^{-}\dot{\theta }\right|\right)dt+\overset{19}{\underset{i=i}{\sum }}\int_{t_{1}}^{t_{2}}\left(\left|F_{i}^{+}\dot{l}\right|+\left|F_{i}^{-}\dot{l}\right|\right)dt & \left(\dot{\theta }\neq 0\right),\\ \underset{\text{agonist}}{\sum }\left|F_{i}^{+}\left(t_{2}-t_{1}\right)\right|+\underset{\text{antagonist}}{\sum }\left|F_{i}^{-}\left(t_{2}-t_{1}\right)\right| & \left(\dot{\theta }=0\right), \end{array}\right. \end{array}$$

where *T*_*i*_^+^ and *T*_*i*_^−^ are the positive and negative joint torques caused by inertia, gravity, and ligament and *F*_*i*_^+^ and *F*_*i*_^−^ are the muscle forces generated by the agonist and antagonist.

The ratio between the mechanical work and the MEE is called mechanical efficiency, which is a significant indicator to evaluate performance of movements in ergonomics:

(12) $$\begin{array}{c} \text{ME}=\frac{\text{W}}{\text{MEE}}, \end{array}$$

### 3.4. Comfort Score of Gestures

The comfort is defined as the level of an operator's well-being when he/she interacts with his/her working environment. Comfortable gesture can greatly relieve fatigue so that operator's working hours can be extended. Different joint angles correspond to different gestures, so ROM of joint is introduced to express the comfort by previous studies [14, 15, 22, 37, 38]. Generally, the ROM is divided into three levels: comfortable range, less comfortable range, and uncomfortable range, and the different levels are assigned different comfort scores. However, the method of ROM is more suitable for comfort evaluation of static gestures, because the quantitative comfort scores are not accurate enough for the whole range of joint motion. In order to obtain more accurate quantitative comfort indicators, the comfort evaluation model is proposed to predict the comfort of gestures based on the MEE of the static and dynamic gestures simultaneously.

The model not only associates the MEE, but also considers the ME as the influence factor of comfort. The comfort score is set between 0 and 10. The higher score indicates a better comfort. The CS corresponding to the MEE and ME can be calculated separately by normalizing as follows:

(13) $$\begin{array}{c} {\text{CS}}_{\text{MEE}}=\frac{\max\left(\text{MEE}\right)-\text{MEE}}{\max\left(\text{MEE}\right)-min\left(\text{MEE}\right)},\\ {\text{CS}}_{\text{ME}}=\frac{\max\left(\text{ME}\right)-\text{ME}}{\max\left(\text{ME}\right)-min\left(\text{ME}\right)}, \end{array}$$

where CS_MEE_ and CS_ME_ represent the comfort scores for MEE and ME, respectively. Thus, the CS can be calculated after assigning different decision weights to the two comfort scores:

(14) $$\begin{array}{c} \text{CS}=w_{1}{\text{CS}}_{\text{MEE}}+w_{2}{\text{CS}}_{\text{ME}}, \end{array}$$

where CS is the comfort score and *w*_1_ and *w*_2_ are weights corresponding to the MEE and ME separately.

## 4. Experiments

**Figure 3:**
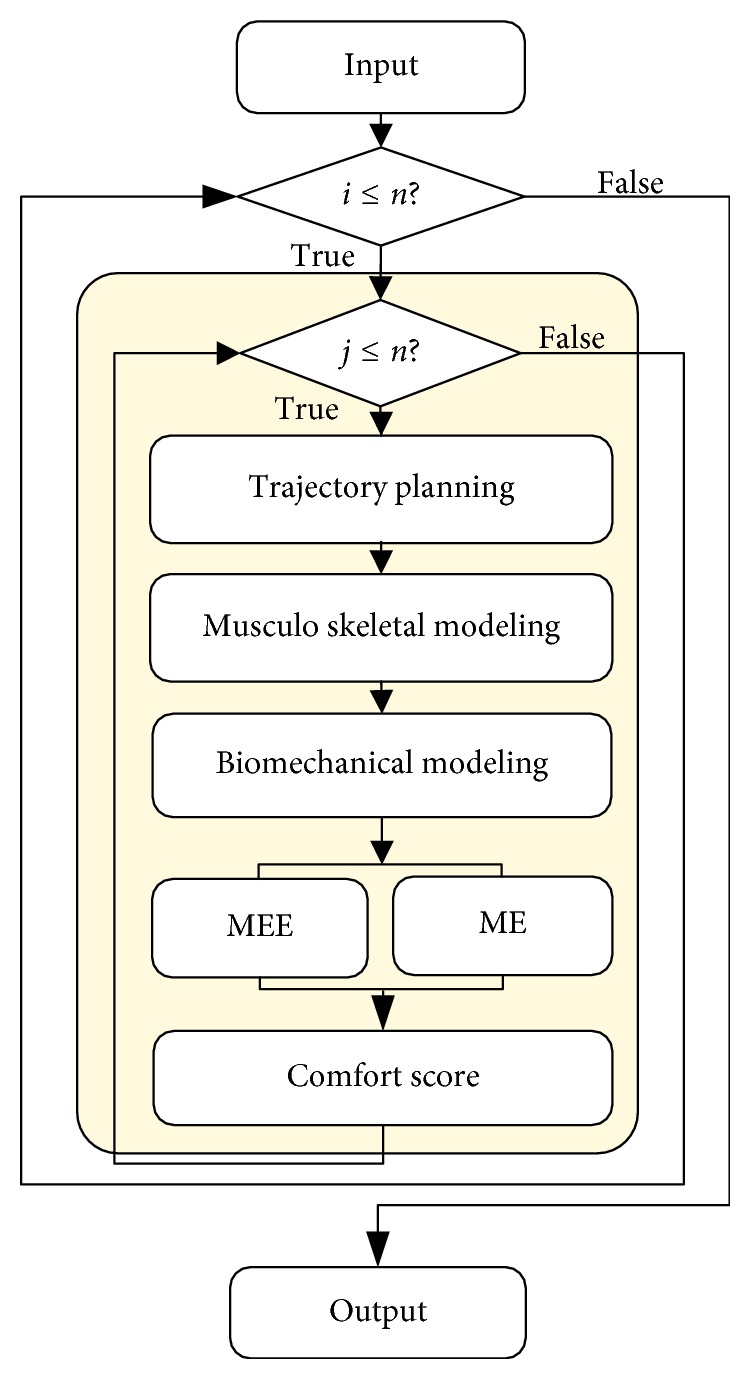
Experiment process represented by flow chart.

The experiments are conducted to simulate the MEE of the human upper limb for predicting the CS. The experiment process is shown in Figure 3. First of all, according to the input parameters, the movements of the upper limb musculoskeletal model are generated by the trajectory planning algorithm in joint space and Cartesian space, respectively. The musculoskeletal model is then applied to simulate during the movements of static and dynamic gestures. After the simulation, the biomechanical model is used to compute the MEE and the ME. Finally, the CS of gestures can be obtained to evaluate the comfort of gestures.

### 4.1. Joint Space Approach

In joint space, the parameter of joint angles (i.e., *θ*=*θ*_1_, *θ*_2_, *θ*_3_, *θ*_4_, *θ*_6_, *θ*_7_) is the input of the model and the trajectories are generated by the trajectory planning algorithm for simulating the human gestures. All the joint angles lie in a reasonable range and satisfy the joint limits.

**Figure 4:**
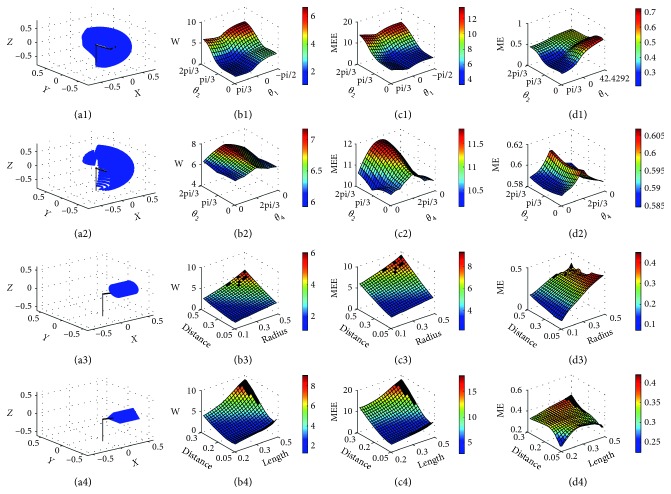
Result of simulation experiments.

As shown in Figure 4(a1), the upper limb model is adopted to represent the gestures that move from the natural sagging state to the upper part of the body. The trajectories consist of gestures with different directions and amplitudes. In joint space, the natural sagging state is set as the initial position, whose parameter of joint angles is *θ*_A_=(*θ*_A1_(*j*), *θ*_A2_, *θ*_A3_, *θ*_A4,_*θ*_A5_, *θ*_A6_, *θ*_A7_). The upper part of the body is a general description that indicates the end position, whose parameter of joint angles is *θ*_B_=(*θ*_B1_(*j*), *θ*_B2_(*i*), *θ*_B3_(*j*), *θ*_B4_(*j*), *θ*_B5_, *θ*_B6_, *θ*_B7_). By changing the parameter of joint angles, the upper limb moves from the initial position to the end position. As shown in Figure 4(a2), the upper limb model simulates the gestures that move from the left side to the right side of the body in different heights and distances. In joint space, the parameter of joint angles of the initial position is *θ*_C_=(*θ*_C1_, *θ*_C2_(*i*), *θ*_A3_, *θ*_C4_(*j*), *θ*_C5_, *θ*_C6_, *θ*_C7_) and the parameter of joint angles of the end position is *θ*_D_=(*θ*_D1_, *θ*_D2_(*i*), *θ*_D3_, *θ*_D4_(*j*), *θ*_D5_, *θ*_D6_, *θ*_D7_). The trajectories represented by the parameters of joint angles are given in Table 3. The joint angles are interpolated into the continuous trajectories by quintic polynomial. The angle velocities and accelerations of initial and end positions are set to zero as the boundary conditions $\left({\dot{\theta }}_{\text{A}/\text{C}}=0,{\dot{\theta }}_{\text{B}/\text{D}}=0,{\ddot{\theta }}_{\text{A}/\text{C}}=0,{\ddot{\theta }}_{\text{B}/\text{D}}=0\right)$. Then, the continuous joint angles, the angel velocities, and the angel accelerations are obtained by the trajectory planning algorithm. Finally, the trajectories in joint space can generate the trajectories in Cartesian space by the forward kinematics.

### 4.2. Cartesian Space Approach

In Cartesian space, the upper limb model is used to simulate the gestures by Cartesian trajectory planning algorithm, and the joint angles can be calculated by the inverse kinematics. The joint angles lie in a reasonable range and satisfy the joint limits.

As shown in Figure 4(a3), the upper limb model is used to represent the human gestures to draw circles in front of the body. The circles have different radius and distances from the center of circle to the body, respectively. The input parameters of the simulation model is (*x*(*i*)*, y, z, r*(*j*)), where the (*x*(*i*)*, y, z*) is the coordinate of the circle center. *x*(*i*) and *r*(*j*) are two variables, and *r*(*j*) represents the radius.

As shown in Figure 4(a4), the upper limb model is used to represent the human gestures to draw equilateral triangles in front of the body. The triangles have different lengths and distances. The input parameters of the simulation model are (*x*(*i*), *y*, *z*, *d*(*j*)), where the (*x*(*i*), *y*, *z*) are the center coordinates of the triangles. *x*(*i*) and *d*(*j*) are two variable, and *d*(*j*) represents the side length of the triangle. The input parameters of trajectory planning algorithm in Cartesian space are given in Table 4.

For the circular trajectories, the coordinates of trajectories in Cartesian space are calculated by using the circular function. The triangle trajectories in Cartesian space are linear and can be fitted by the quintic polynomial. Finally, the Cartesian trajectories can be used to create joint trajectories by using the inverse kinematics.

## 5. Results and Discussion

### 5.1. Results

The MEE consists of the muscle mechanical energy, the kinematic energy, the gravitational potential energy, and the energy expenditure of the ligament. The upper limb model is created to establish the link between the comfort of gestures and the MEE of both static and dynamic gestures. The work of gestures, the MEE, and the ME are calculated for each gesture in experiments, and the results are shown in Figure 4.

Firstly, gestures of each experiment can form various trajectories that consist of four hundred trajectories with different parameters. The trajectories of gestures are visualized in the three-dimensional space, as shown in Figures 4(a1)–4(a4). In Figure 4(a1), the trajectories from the bottom to the top composed of different directions and heights can be obtained by changing the joint angles *θ*_1_ and *θ*_2_. In Figure 4(a2), the trajectories from the left to the right composed of different heights and distances can be obtained by changing the joint angles *θ*_2_ and *θ*_4_. Figure 4(a3) shows the circular trajectories that are obtained by changing the circular radius *r*(*j*) and the distance from the body to the center of the circles *x*(*i*). Figure 4(a3) shows the triangle trajectories that are obtained by changing the side length of the triangle *d*(*j*) and the distance from the body to the center of the triangle *x*(*i*).

Figures 4(b1)–4(b4) show the work of gestures, respectively, and the results also reveal the distribution regularities of the work of gestures along different trajectories. Figures 4(c1)–4(c4) reveal the MEE distribution regularities of gestures along different trajectories. Figures 4(d1)–4(d4) reveal the distribution regularities of the ME along different trajectories. The coordinates *x* and *y* correspond to the input parameters and the coordinates *z* corresponds to the values of the work of gestures, the MEE, and the ME with different colors. In Figures 4(c3), 4(d3), 4(c4), and 4(d4), the black areas represent the singular positions, where the muscle strengths are too large and the joint's limit is exceeded.

**Figure 5:**
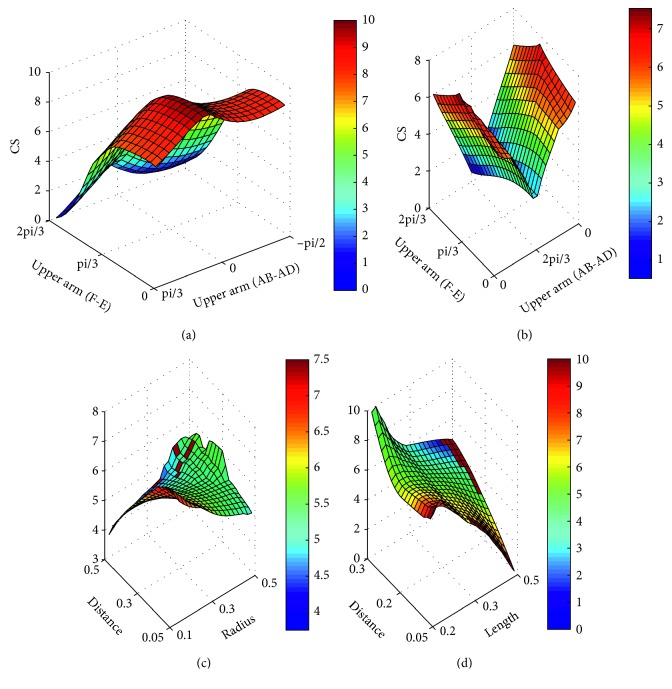
Comfort score of gestures.

The MEE and the ME contain rich biomechanical information, and thus, both of them can be used to predict the ergonomic level of the gestures. The CS is calculated based on the MEE and the ME to evaluate the comfort of gestures. As shown in Figure 5, the CS is set between 0 and 10. A higher score indicates a better comfort. The black areas are the singular positions that demonstrate an unfavorable ergonomic level.

### 5.2. Comparisons

**Figure 6:**
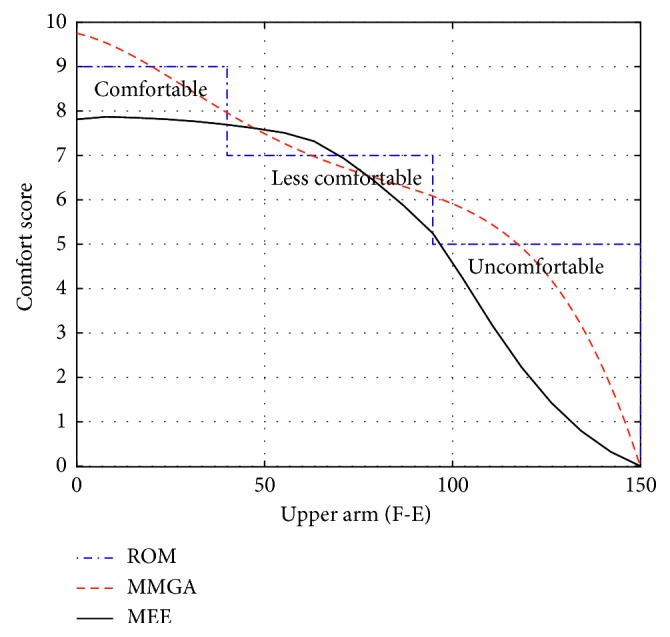
Comparison of F-E joint of upper arm.

**Figure 7:**
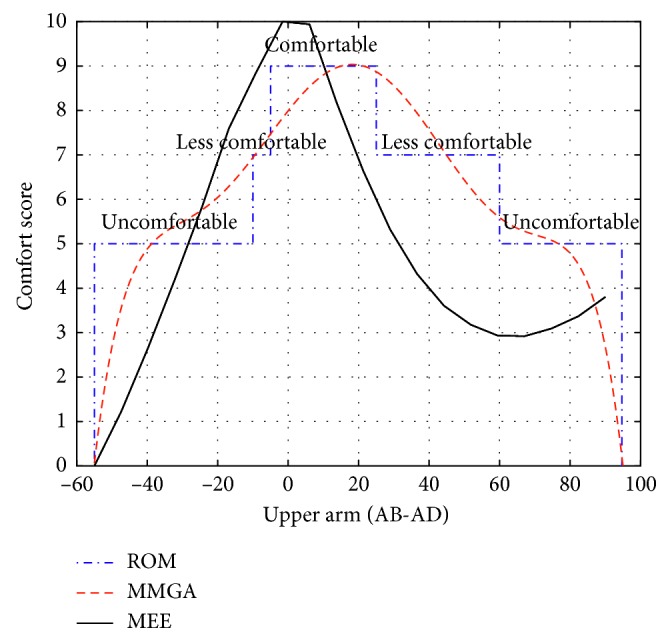
Comparison of AB-AD joint of upper arm.

**Figure 8:**
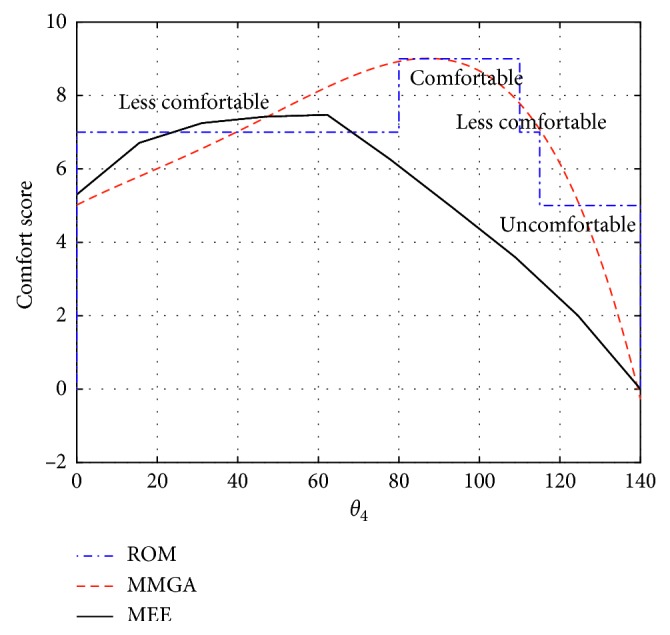
Comparison of F-E joint of forearm.

For a better understanding of the differences among the comfort evaluation models based on the ROM, the MMGA, and the MEE, the comparison results are shown in Figures 6–8. Three joint angles are chosen to demonstrate the differences.

The ROM of flexion-extension joint of upper arm is divided into three parts. According to simulation result obtained from the model based on the ROM, the ROM from 0° to 35° is comfortable, the ROM from 35° to 90° is less comfortable, and the ROM from 90° to 150° is uncomfortable, which are represented by the blue dotted lines in Figure 6. The black curve in Figure 6 represents the comfort score of gestures from 0° to 150° obtained by the proposed evaluation model. The model based on the MMGA provides a quantitative value for the ergonomic ranking, and the comfort indicator for each joint is computed through a spline fitting of the comfort ranks derived from the LUBA method along the ROM of joints. The comparison result shows both the consistency and the difference between the model based on the MEE with those based on the ROM and the MMGA. The gesture with a higher CS is located in the comfortable range while the gesture with a lower CS is located in the uncomfortable range.

In Figure 7, the ROM of abduction-adduction joint of upper arm is divided into three parts from −55° to 90°. The ROM from −5° to 25° is comfortable, the ROM from −20° to −5° and from 25° to 60° are less comfortable, and the ROM from 60° to 90° is uncomfortable. A slight difference can be found between the curves which represents the comfort score and the three comfort levels obtained by the model based on the ROM.

Figure 8 shows the comparison of flexion-extension joint of forearm that is divided into three parts from 0° to 140°. The ROM from 80° to 110° is comfortable, the ROM from 0° to 80° and from 110° to 115° are less comfortable, and the ROM from 115° to 140° is uncomfortable. Even though the general trend of the CS obtained from the model based on the MEE keeps consistent with the comfort levels based on the ROM and the MMGA, the difference among the comfort evaluation models based on the ROM, the MMGA, and the MEE can be also found in Figure 8 (e.g., the gesture with the highest CS is not located in the comfortable range of joint). The reasons for the differences among the ROM, MMAG, and MEE will be discussed in the following sections.

### 5.3. Discussion

The purpose of the paper is to develop a comfort evaluation model to predict the comfort gestures, which considers both static and dynamic gestures. The inertial force, the centrifugal force, the gravity force, the muscle force, and the limit of ligaments to the joint are considered as the factors which affect the comfort of gestures. The results can provide an effective support to predict the risk of fatigue and injury for joints and muscles.

As shown in Figures 4(b1), 4(c1), and 4(d1) and Figures 4(b2), 4(c2), and 4(d2), the relationships between the work of gestures, the MEE, and the ME with the joint angle are revealed. The flexion-extension joint of the shoulder has more impact on the work of gestures and MEE than the Abduction-Adduction joint, but has less impact on the ME than Abduction-Adduction joint. Figures 4(b3), 4(c3), and 4(d3) and Figures 4(b4), 4(c4), and 4(d4) show the relationships between the distributions of the work of gestures, the MEE, and the ME with the distance *x*, the side length *d*, and the radius *r*. The distance has more impact on the work of gestures and the MEE than the side length and the radius, but has less impact on the ME than the side length and the radius. The black areas represent the gestures that cause joint damage or muscle fatigue, because the muscle strengths are too large and the joint's limit is exceeded. The results help designers to understand the biomechanical properties of the upper limb during the movements. Moreover, the results offer a quantitative and intuitive indicator to predict which act has the higher risk of fatigue or injury for joints and muscles.

Figure 5 shows the relationships between the comfort scores with the joint angle, the distance, the side length, and the radius. The black areas represent the gestures with unfavorable ergonomic level. In other words, the gestures in black areas should be avoided during the gesture interaction, because the joint has already attained its limit positions, which causes the joint damage and the muscle fatigue consequently. The results provide a quantitative indicator for the comfort of gestures and predict the risk of joint damage and muscle fatigue.

The comparisons among the comfort evaluation models based on the ROM, the MMGA, and the MEE are shown in Figures 6–8. The comfort evaluation model based on the ROM aims at the comfort evaluation of static gestures. The comfort evaluation model based on the MMGA intends to predict the comfort of dynamic gestures through a spline fitting of the comfort ranks for static gestures, but the relative kinematic characteristics during the movements of dynamic gestures, such as arm's gravity and its inertia, the centrifugal force, the muscle force, and the limit of ligaments to the joint, are not taken into consideration. The proposed comfort evaluation model can account for the static and dynamic gesture from the perspective of MEE. The consistency among the flexion-extension simulation result of comfort score and that based on the ROM and the MMGA for the upper arm is shown in Figure 6. However, there exist differences between the abduction-adduction (or the flexion-extension) simulation result of CS and that based on the ROM and the MMGA for the upper arm (or forearm) (Figures 7 and 8).

Differences can be found while comparing the proposed comfort evaluation model based on MEE with the models based on ROM and MMGA. One major reason for such differences is the different modeling principles applied on the three evaluation models. The comfort scores from the models based on ROM considers only the static gestures; however, the proposed comfort evaluation model based on MEE considers the influence of both static and dynamic gestures. In other words, the comfort scores obtained from the model based on ROM represent the comfort levels of static postures, whereas the comfort scores of the model based on the MEE describe the comfort levels of dynamic actions. As for the model based on the MMGA, although it intends to consider the dynamic gestures, the comfort of dynamic gestures is predicated through a spline fitting of the comfort ranks for static gestures, and the arm's gravity and its inertia, the centrifugal force, the muscle force, and the limit of ligaments to the joint during the movements of dynamic gesture are not taken into consideration.

## 6. Conclusion

The paper proposes a musculoskeletal biomechanical model based on the mechanical energy expenditure and the mechanical efficiency to predict the comfort of both static and dynamic gestures. On the one hand, the proposed comfort evaluation model supports the quantitative measure of comfort of gestures without using the electromyography or other measuring devices; on the other hand, from the ergonomic perspective, it provides an intuitive indicator to predict which act has the higher risk of fatigue or injury for joints and muscles, so as to reduce operators' fatigue and extend their working hours. Experiments are then conducted to validate the effectiveness of the proposed model. The comparison result shows both the consistency and the difference between the model based on the MEE with those based on the ROM and the MMGA. The reasons for the difference are the ignorance of dynamic gestures in the model based on the ROM and the ignorance of kinematic characteristics during the movements of dynamic gesture in the model based on the MMGA.

In the future, the authors will adopt statistical methods, such as the statistical significance tests, to analyze the differences between the proposed evaluation model and those based on the ROM and MMGA. Furthermore, the research will be applied on the optimization of gesture design while considering the feedbacks of perceived effects and gesture recognition rate in HCI, so that the comfortable, efficient, and human-computer friendly interaction gestures can be achieved.

## Figures and Tables

**Table 1:** Advantages and limitations for comfort evaluation models.

Model	Study	Advantage	Limitations
(i) Based on ROM	RULA [10]	(i) Available for static gestures (ii) Simple for modeling (iii) Fast calculation (iv) No equipment (v) Convenient (vi) Need less professional skills	(i) Not available for dynamic gesture (ii) Discontinuous indicator (iii) Less precise quantitative value for ergonomics (iv) Less biomechanical information
	LUBA [12]		
	REBA [13]		
	NERPA [14]		
	UNE-EN 1005 [14]		
	OCRA [19]		

(ii) Based on ROM and movement data	Chen [20]	(i) Available for static and dynamic gestures (ii) Fast calculation (iii) No equipment (iv) Partial biomechanical information (v) Continuous indicator	(i) Less precise quantitative value for ergonomics (ii) Complex model (iii) Need certain professional skills
	MMGA [21]		
	Weede [23]		
	DHM [24]		
	Battini [31]		

(iii) Based on software	JACK [15]	(i) Precise quantitative value for ergonomics (ii) Continuous indicator (iii) Convenient	(i) Need more professional skills
	CATIA [25]		

(iv) Based on sensors	Mocap [26]	(i) More precise quantitative value for ergonomics (ii) Rich biomechanical information (iii) Continuous indicator	(i) Need equipment (ii) Need more professional skills (iii) Time consuming (iv) Costly
	EMG [27]		

**Table 2:** Relationship between muscles and joint motions.

Muscle	Joint	Movement
Latissimus dorsi (*l*_1_)	Shoulder	Flexion/extension
Coracobrachialis (*l*_2_)		
Supraspinatus (*l*_3_)		Adduction/abduction
Pectoralis major (*l*_4_		
Posterior Deltoid (*l*_5_)		
Teres major (*l*_6_)		
Subscapularis (*l*_7_)		Pronators/supinators
Teres Minor (*l*_8_)		
Biceps Brachii (*l*_9_)	Shoulder/Elbow	Flexion/extension
Triceps Brachii (*l*_10_)		Flexion/extension
Anconeus (*l*_11_)	Elbow	Flexion/extension
Brachioradialis (*l*_12_)		
Supinator (*l*_13_)		Pronation/supination
Teretipronator (*l*_14_)		
Flexor carpi ulnaris (*l*_15_)	Elbow/Wrist	Flexion/extension
Extensor carpi ulnaris (*l*_16_)	Wrist	Flexion/extension
Flexor carpi radialis (*l*_17_)		
Extensor carpi ulnaris (*l*_18_)		Adduction/abduction
Flexor carpi ulnaris (*l*_19_)		

**Table 3:** Trajectories represented by parameters of joint angles in joint space.

Input	*θ*_A_	*θ*_B_	*θ*_C_	*θ*_D_
*θ*_1_	[60°, −90°]	[60°, −90°]	45°	−90°
*θ*_2_	0°	[0°, 150°]	[10°, −60°]	[10°, −60°]
*θ*_3_	0°	[30°, 0°]	0°	0°
*θ*_4_	0°	[120°, 60°]	[0°, −90°,0°]	[0°, −90°, 0°]
*θ*_5_	0°	90°	0°	0°
*θ*_6_	0°	−30°	10°	0°
*θ*_7_	0°	0°	0°	0°

**Table 4:** Input parameters in Cartesians space (unit: m).

Input	*x*(*i*)	*y*	*z*	*r*(*j*)/*d*(*j*)
Circle	[0.25, 0.5]	−0.185	0	[0.05, 0.2]
Triangle	[0.25, 0.5]	−0.185	0	[0.05, 0.3]

## Data Availability

The data used to support the finding of this study (trajectory planning of upper limb motion) are included in the article (Tables 3 and 4). Previously reported data related to the ROM of joints were used to compare with the results of this study and are available at (doi: https://doi.org/10.1155/2015/896072). This prior study is cited at a relevant place within the text as references [16]. Previously reported data of comfort evaluation model based on the MMGA were used to compare with the results of this study and are available at (doi: https://doi.org/10.1007/978-3-642-02809-0_62). This prior study is cited at relevant place within the text as references [22].
